# Prevalence and Imaging Correlates of Cerebral Diaschisis After Ischemic Stroke: A Systematic Review and Meta-Analysis

**DOI:** 10.3390/brainsci16010050

**Published:** 2025-12-29

**Authors:** Qi Jia, Nannan Sheng, Gilles Naeije

**Affiliations:** Neurology Department, Hôpital Universitaires de Bruxelles-Hôpital Erasme, 808 route de Lennik, 1070 Bruxelles, Belgium; qi.jia@ulb.be (Q.J.); nannan.sheng@ulb.be (N.S.)

**Keywords:** ischemic stroke, diaschisis, functional disconnection, neuroimaging techniques, prevalence

## Abstract

**Background/Objectives:** Diaschisis, reduced neural activity, perfusion, and metabolism in structurally intact but anatomically connected regions, is a network-level consequence of focal brain injury. Despite the extensive literature, its prevalence across imaging modalities and diaschisis subtypes has not been systematically synthesized. This review aims to identify convergent evidence for diaschisis after ischemic stroke and clarify how its detection relates to neuroanatomical disconnection, clinical factors, and imaging methods. (PROSPERO: CRD420251017909). **Methods:** PubMed and Embase were searched through February 2025 for studies reporting quantitative measures of diaschisis using perfusion, metabolic, or functional imaging. Pooled prevalence and modality-specific estimates were calculated. Subgroup analyses examined diaschisis subtypes, stroke severity, age, and study quality. **Results:** Sixty-six studies (3021 patients) were included. Overall pooled prevalence was 53% (95% CI: 47–58%). Crossed cerebellar diaschisis was most frequently studied (49%), while thalamic and other remote patterns showed comparable or higher effect sizes. Detection varied primarily by imaging modality: ASL MRI (67%) and PET (58%) showed the highest sensitivity; SPECT (53%) and CTP (49%) were intermediate; DSC-PWI had the lowest (28%). In contrast, age had no measurable effect and stroke severity only modestly increased detection, suggesting that diaschisis is driven predominantly by neuroanatomical disconnection rather than demographic or clinical variables. Egger’s tests indicated minimal publication bias. **Conclusions:** Diaschisis is a common manifestation of network vulnerability after ischemic stroke, determined chiefly by lesion topology and long-range anatomical connectivity. Detection depends more on imaging physiology than patient characteristics. Standardized definitions and longitudinal multimodal studies are needed to clarify its temporal evolution and clinical significance.

## 1. Introduction

**Figure 1:**
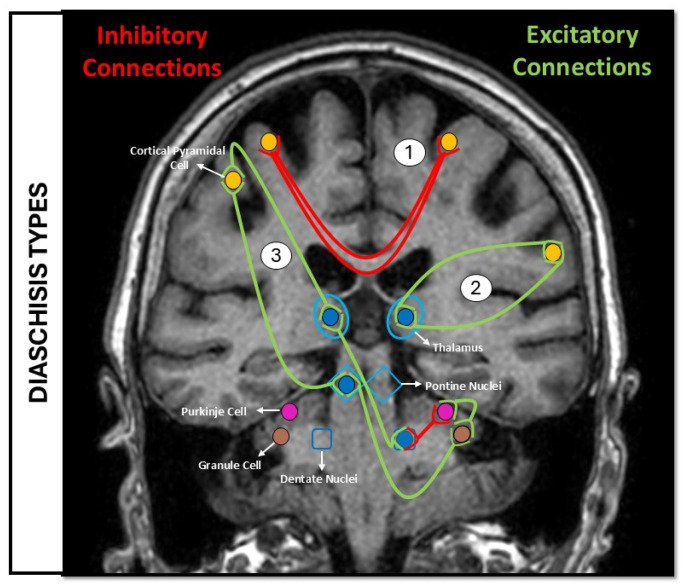
Schematic illustration of the main neural pathways involved in the different types of diaschisis. Green lines: excitatory connections; red lines: inhibitory connections. (1) Interhemispheric diaschisis: loss of inhibition to contralesional motor cortex; (2) thalamo-cortical diaschisis: ipsilateral loss of thalamic excitation by neocortical areas; and (3) crossed cerebellar diaschisis (CCD): loss of excitatory cerebellar afferences from contralateral cortical areas.

The phenomenon of diaschisis was first described by Von Monakow in 1914, meaning that a reduction in neural activity, blood perfusion (cBF), or metabolic function in intact but functionally connected regions remote from a focal brain lesion [1,2]. Diaschisis has become a key concept for understanding the network-level consequences of focal injury. Rather than representing structural damage, diaschisis reflects secondary functional depression caused by disruption of long-range anatomical pathways, including corticocerebellar, thalamocortical, and cortico-subcortical circuits [3,4]. In stroke, particularly in ischemic stroke, transhemispheric, thalamic, and cerebellar diaschisis [5,6,7] have been described at both acute and chronic stages (Figure 1), this remote dysfunction may influence both early neurological deficits and later recovery, making it a phenomenon of considerable interest in clinical neuroscience. Despite its longstanding recognition, the reported prevalence of diaschisis varies widely across studies. This variability reflects differences in patient populations, timing after stroke, imaging protocols, and definitions of hypoperfusion or hypometabolism. Moreover, diaschisis manifests in multiple forms—including crossed cerebellar, thalamic, cortical, and transhemispheric subtypes—each associated with distinct neuroanatomical connections. Among these, crossed cerebellar diaschisis (CCD) is the most commonly described after hemispheric stroke, typically presenting as reduced perfusion and metabolism in the cerebellar hemisphere contralateral to a supralateral infarct [8]. For the purpose of this meta-analysis, diaschisis was operationally defined as a reduction in perfusion, metabolism, or neural activity occurring in a structurally intact brain region that is anatomically connected to, but spatially remote from, a focal ischemic lesion. This definition emphasizes network-mediated functional depression rather than vascular contiguity. Within this framework, crossed cerebellar diaschisis represents the prototypical form of ‘classical’ network diaschisis, whereas other remote patterns (e.g., thalamic or transhemispheric hypoperfusion) may reflect partially overlapping but potentially heterogeneous mechanisms of disconnection.

With advances in neuroimaging, the detection of diaschisis has become increasingly diverse. Numerous imaging techniques have been used to measure these changes, such as Positron Emission Tomography (PET), Single-photon Emission Computed Tomography (SPECT), Arterial Spin Labeling MRI (ASL), and Computed Tomography Perfusion imaging (CT Perfusion), and diffusion-or perfusion-weighted MRI (DWI/PWI-MRI). Broadly, these imaging techniques used to evaluate diaschisis can be categorized into two groups. (i) perfusion-based methods that detect diaschisis by revealing reductions in cerebral blood flow or perfusion in structurally intact but functionally deactivated brain regions, reflecting hemodynamic consequences of disrupted neural connectivity following a distant lesion [9]; and (ii) metabolism-based methods revealing diaschisis by detecting hypometabolism in structurally intact brain regions functionally disconnected from the lesion, reflecting reduced neuronal activity [10]. Each technique captures a different aspect of remote physiological depression, the sensitivity for detecting diaschisis differs among various neuroimaging modalities, and the reported prevalence rates of diaschisis vary widely across studies, ranging from 16% for CCD with DSC-PWI [11] to 91% for CCD with SPECT [12] in hemispheric strokes. However, there has been no integrated synthesis evaluating how these modalities collectively characterize diaschisis across the spectrum of ischemic stroke. Previous studies typically focus on single modalities or isolated subtypes, limiting our understanding of diaschisis as a transmodal network phenomenon. Furthermore, although many articles describe diaschisis, the literature has become fragmented, with inconsistent terminology and heterogeneous methodological criteria.

These considerations underscore the need for a systematic, cross-modal evaluation to determine how the various imaging techniques converge in their depiction of diaschisis. Importantly, the aim will not be to compare techniques or determine the “best” method for detecting diaschisis. Instead, the goal will be to identify converging evidence across modalities that measure the same underlying physiological process: remote functional depression secondary to focal ischemic injury. Mapping the prevalence and distribution of diaschisis across imaging types will help clarify its general characteristics, inform its neurobiological interpretation, and guide future research towards standardized definitions and clinically meaningful applications. Accordingly, the present systematic review and meta-analysis (i) will synthesize evidence on the prevalence of diaschisis across ischemic stroke studies, (ii) compare how different imaging modalities detect diaschisis as a remote dysfunction phenomenon, and (iii) characterize the distribution of diaschisis subtypes across lesion locations and disease stage. By integrating results across decades of perfusion, metabolic, and functional imaging research, this work aims to provide the most comprehensive overview to date of diaschisis after ischemic stroke and to establish a framework for future studies on its mechanisms, temporal evolution, and clinical relevance.

## 2. Methods

### 2.1. Study Design and Registration

This systematic review and meta-analysis was conducted in accordance with the Preferred Reporting Items for Systematic Reviews and Meta-Analyses (PRISMA 2020) guidelines [13]. The study protocol was prospectively registered in the International Prospective Register of Systematic Reviews (PROSPERO) under the registration number CRD420251017909 (see Supplementary Material S1).

### 2.2. Literature Search Strategy

A comprehensive search was performed in PubMed and Embase from inception to February 2025. The search strategy incorporated both free-text terms and MeSH terms, including “Stroke,” “Diaschisis,” and “Functional disconnection” (see full strategy in Supplementary Material S2). Additionally, we performed manual backward and forward citation tracking of all included articles and relevant reviews to identify further eligible studies. Two independent reviewers (Q.J. and N.G.) screened records using EndNote 9.0 to remove duplicates and performed a two-level screening via Covidence. The screening process included the following: (i) Exclusion of non-original studies such as reviews, case reports, letters, and conference abstracts. (ii) Title and abstract screening. (iii) Full-text review to confirm availability of data on diaschisis prevalence or sufficient information to calculate it. Disagreements were resolved by discussion or consultation with a third reviewer.

### 2.3. Eligibility Criteria

Inclusion criteria:

(1) Population: Studies involving patients with ischemic stroke who did not undergo decompressive hemicraniectomy or other open neurosurgical intervention to avoid perfusion/metabolic changes unrelated to classical diaschisis.
(2) Outcome/Definition: Studies must report diaschisis or provide sufficient information to derive diaschisis prevalence. For the purpose of this meta-analysis, we defined diaschisis as a remote reduction in perfusion, metabolism, or regional activity in a structurally intact brain region that is anatomically connected to the index lesion. Studies were eligible if they either (i) explicitly labeled the finding as diaschisis (e.g., CCD, thalamic, transhemispheric) or (ii) reported regional hypoperfusion/hypometabolism/reduced regional activation in a remote but structurally preserved region such that a reviewer could reasonably classify it as diaschisis according to the above definition.
(3) Imaging modality: First, perfusion-based methods, including the following: (i) CT Perfusion, which measures cerebral blood flow (CBF), cerebral blood volume (CBV), and Time to maximum (Tmax), providing insight into hemodynamic changes associated with ischemia [14]. (ii) SPECT, which assesses regional perfusion by detecting the uptake of radiotracers, indirectly reflecting cerebral blood flow from a static perspective [15]. (iii) Magnetic Resonance Imaging (MRI) (including ASL and DSC-PWI): ASL-MRI is a non-invasion perfusion technique that uses magnetically labeled arterial blood water as an endogenous tracer to assess CBF [16]. DSC-PWI is a dynamic MRI perfusion method that uses gadolinium contrast, measures perfusion parameters similar to CTP, but with higher spatial resolution [17]. Second, metabolism-based methods composed of (i) PET using, including ^18^FDG-PET or C^15^O_2_-PET [18], and (ii) Functional MRI [19].
(4) Study design and data: Original research articles that report the numerator (number of patients with diaschisis) and denominator (total number of patients assessed or assessable) or provide sufficient data to derive prevalence.
(5) Language and accessibility: Studies published in English (and other languages if data is complete) with full text available.
(6) Although BOLD-fMRI does not directly quantify perfusion or metabolic rate, it reflects neurovascular coupling and regional neural activity. fMRI studies were therefore included only when they demonstrated reduced signal or activation in remote, structurally preserved regions consistent with functional depression secondary to anatomical disconnection. Pure connectivity-based analyses without evidence of regional suppression were excluded. This approach allowed complementary physiological measures to converge on a shared network-level construct.

Exclusion criteria:

(1) Studies not focused on diaschisis or functional disconnection and do not provide extractable prevalence data or insufficient information to derive the numerator/denominator.
(2) Patients undergoing decompressive hemicraniectomy or other open neurosurgical procedures, because postoperative perfusion/metabolic changes do not reflect classical diaschisis.
(3) Studies involving non-ischemic conditions such as hemorrhagic stroke or brain tumors, or animal and in vitro studies.
(4) Studies that only reported connectivity metrics (e.g., functional connectivity correlations) without reporting regional hypoperfusion/hypometabolism/reduced activation in a specific remote region.
(5) Studies that describe regional low perfusion/metabolism, but the affected region is anatomically contiguous with the infarct (i.e., not “remote”), or where remote status/structural integrity cannot be established from the text/figures.
(6) Studies where the low perfusion/metabolism is due to a separate primary pathology, unless diaschisis is explicitly demonstrated as secondary to an acute focal ischemic event.
(7) Non-English-language publications without quantitative data; meeting abstracts; commentaries; letters; or case reports.

### 2.4. Data Extraction

Two reviewers independently extracted data and cross-checked for accuracy. Extracted data included the following: (1) General study information (author, year, country, region, study design). (2) Patient characteristics (sample size, age, sex, stroke phase, NIHSS score, occluded vessel type). (3) Imaging modality used to assess diaschisis. (4) Diaschisis subtype (CCD, ITD, or others) as reported by each study. (5) Follow-up data and time points (if available). (6) Study quality (NOS score). (7) Outcomes: Number of patients with diaschisis, total sample size, and calculated prevalence.

### 2.5. Quality Assessment

The quality of included studies was assessed using the Newcastle–Ottawa Scale (NOS) for non-randomized studies in meta-analyses [20], which evaluates three domains, selection (4 items), comparability (1 item), and outcome/exposure (3 items), for a maximum score of 9. A score of ≥7 was defined as high quality, and <7 as lower quality. Two reviewers scored independently; disagreements were resolved through discussion. Studies with scores < 4 were excluded.

### 2.6. Subgroup and Sensitivity Analysis

Because standardized clinical cut-offs for age and stroke severity were not consistently reported across studies, subgroup thresholds were defined using a data-driven approach based on pooled mean values across the dataset (<63 vs. ≥63 years for age; <8.8 vs. ≥8.8 for NIHSS). These thresholds were used for exploratory purposes to investigate sources of heterogeneity rather than to imply clinically prescriptive categories. While these thresholds do not correspond to established clinical categories, their use is consistent with recommendations for exploratory analyses when predefined criteria are unavailable [21,22,23,24]. This strategy was adopted to investigate potential sources of heterogeneity in the reported prevalence of diaschisis. Subgroup analyses were conducted according to the following variables: (1) imaging modality (CTP, SPECT, ASL MRI, DSC-PWI, XeCT, PET, fMRI); (2) diaschisis subtype, including crossed cerebellar diaschisis (CCD), no CCD for both ipsilateral thalamic diaschisis (ITD), and others; (3) NIHSS score category; (4) age group; and (5) study quality, assessed using the Newcastle–Ottawa Scale (NOS ≥ 7 vs. <7). Only first-time imaging data were included from each study. In cases where multiple types of diaschisis were reported, CCD data were prioritized to ensure consistency across analyses [22].

### 2.7. Follow-up Studies

Six studies reported longitudinal assessment of diaschisis [25,26,27,28,29,30]. Due to wide variation in follow-up duration (ranging from 3 days to 5 years), only descriptive analysis and forest plot visualization were performed. These studies were excluded from pooled meta-analysis.

### 2.8. Publication Bias Assessment

Publication bias was assessed via funnel plots and Egger’s test, as recommended by the PRISMA guidelines and Cochrane Handbook for systematic reviews [31]. Egger’s test was conducted only for subgroups with ≥10 studies [32]. A *p*-value < 0.05 indicated significant bias; 0.05–0.1 indicated possible bias; and ≥0.1 suggested no evidence of bias.

### 2.9. Statistical Analysis

Meta-analysis was conducted using Stata 18.0. The primary outcome was the prevalence of diaschisis. For I^2^ > 50% [33] or when significant clinical/methodological heterogeneity was suspected, the DerSimonian–Laird random-effects model was used to account for potential heterogeneity across studies. This approach assumes that true prevalence estimates vary across studies, and provides an estimate of the mean prevalence across a distribution of effects rather than a single common value, which would be implausible in this context [34]. High between-study heterogeneity (I^2^ frequently > 90%) was anticipated due to the broad scope of the literature, encompassing different imaging physiologies (perfusion-, metabolism-, and activation-based methods), heterogeneous stroke populations, and variable timing of assessment. In this context, heterogeneity reflects real differences in study-level effects rather than methodological error. For subgroups with I^2^ = 0 and consistent design, a fixed-effect model was applied to improve estimate precision. The results are presented as pooled prevalence with 95% confidence intervals (CIs). Forest plots were generated to visualize effect sizes across studies. To further explore potential outliers and sources of heterogeneity, Galbraith plots were used for sensitivity analysis [35]. All statistical tests were two-sided with a significance threshold of *p* < 0.05. Each subgroup meta-analysis was also conducted using the DerSimonian–Laird random-effects model to account for between-study variability. Effect sizes were expressed as standardized mean differences (SMDs) with 95% confidence intervals (CIs), and heterogeneity was quantified using the I^2^ statistic. Statistical significance was set at a two-sided *p*-value < 0.05. Interpretation of SMDs followed Cohen’s conventional benchmarks, where values around 0.2, 0.5, and 0.8 represent small, medium, and large effect sizes, respectively.

## 3. Results

### 3.1. Literature Search and Study Selection

**Figure 2:**
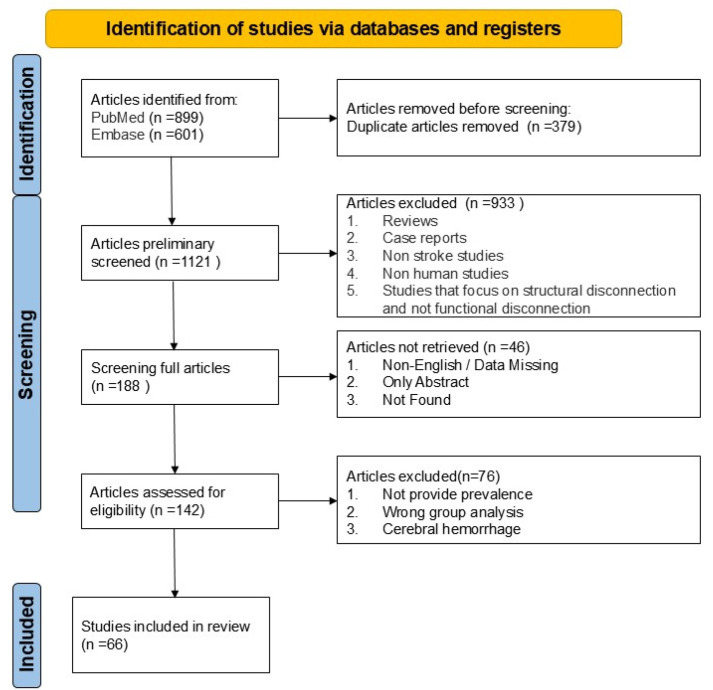
PRISMA flow diagram for study selection in the meta-analysis of diaschisis prevalence in ischemic stroke.

A total of 1500 articles were identified through initial searches, including 899 from PubMed and 601 from Embase. After removing 379 duplicates using EndNote software 2025, 1121 records remained. Two reviewers (N.G. and Q.J.) screened titles and abstracts in Covidence. A total of 933 articles were excluded for reasons including: reviews, case reports, non-stroke populations, non-human studies, and studies on structural rather than functional disconnection. After initial screening, 188 full-text articles were assessed for eligibility. Of these, 46 were excluded due to unavailability (18 non-English articles and lacking sufficient data for calculation of diaschisis prevalence, 22 with abstracts only, 6 not retrievable). An additional 76 studies were excluded for reasons including missing prevalence data, inappropriate population (e.g., hemorrhagic stroke or tumor), or analytical issues. Ultimately, 66 studies met the inclusion criteria and were included in the meta-analysis. The study selection process is summarized in the PRISMA flow diagram (Figure 2). The main characteristics of all included studies are summarized in Supplementary Table S1.

### 3.2. Study Population and Characteristics

A total of sixty-six studies involving 3021 adult patients with ischemic stroke were included. Among them, 1319 were male and 947 were female; ten studies (*n* = 755) did not report sex distribution; eight studies did not report age data, and the mean age across studies was approximately 63 years (Supplementary Table S1). All participants were ≥18 years old. Some studies reported age as mean ± standard deviation, while others provided medians and interquartile ranges, which were approximated using the formula (Q1 + 2 Median + Q3)/4 [36].

### 3.3. Study Design and Geographic Distribution

Based on study design, the included studies were classified as follows: cross-sectional studies: 37; retrospective cohort studies: 19; case–control studies: 8; prospective cohort studies: 2. In terms of geographic distribution: Europe: 36 studies; Asia: 21 studies (China: 10; Japan: 8; South Korea: 3); North America: 9 studies (United States: 8; Canada: 1). See Supplementary Table S1.

### 3.4. Imaging Modalities and Types of Diaschisis

Seven types of neuroimaging techniques were used among the included studies: CT Perfusion (CTP): ten studies; SPECT: thirty studies; PET: fourteen studies; ASL MRI: four studies; DSC-PWI: three studies; fMRI: three studies; XeCT: two studies. Regarding the types of diaschisis reported: fifty-one studies reported only crossed cerebellar diaschisis (CCD); one study reported only ipsilateral thalamic diaschisis (ITD); six studies reported both CCD and ITD; and eight studies included other types, such as transhemispheric diaschisis, cerebello-cerebral diaschisis, ipsilateral cerebellar diaschisis, and ipsilateral cortical diaschisis. See Supplementary Table S1.

### 3.5. Occluded Vessel Types and Stroke Phases

According to the type of occluded vessels, 41 studies involved large-vessel strokes (e.g., ICA, MCA, ACA), 10 studies involved small-vessel strokes (e.g., thalamic, cortical, or internal capsule territories), and 15 studies did not specify vessel types. Stroke phases were classified based on the staging criteria proposed by Julie Bernhardt et al. [37] (0–24 h: hyperacute; 24 h–7 days: acute; 7 days–3 months: early subacute; 3–6 months: late subacute; >6 months: chronic). For simplicity, this study grouped stroke phases as follows: acute phase (≤7 days): 26 studies; subacute/chronic phase (>7 days): 26 studies; unclassifiable (due to broad or unclear time range): 14 studies. See Supplementary Table S1.

### 3.6. NIHSS Scores and Follow-up Status

Among the 63 included studies, 16 studies provided NIHSS scores at admission, with an average NIHSS score of 8.8. The remaining studies did not report NIHSS data. In terms of follow-up, six studies reported longitudinal diaschisis imaging during follow-up. However, the follow-up intervals varied significantly (ranging from 3 days to 5 years). Due to this heterogeneity, these studies were analyzed descriptively and were not included in the pooled meta-analysis. See Supplementary Table S1.

### 3.7. Risk of Bias and Quality Assessment

All 66 included studies were evaluated using the Newcastle–Ottawa Scale (NOS), with scores ranging from 4 to 9 and an average score of 6.6. The quality distribution was as follows: high-quality studies (NOS ≥ 7): 33; moderate-quality studies (NOS 4–6): 33; low-quality studies (NOS < 4): 0 (none were included). See Supplementary Table S1. By study type, the average NOS scores were as follows: prospective cohort studies: 7.5 (highest); retrospective cohort studies: 6.7, cross-sectional studies: 6.5; case–control studies: 6.3 (lowest). Some studies lacked important clinical information, occluded vessel type, stroke onset-to-imaging time window, or NIHSS scores. These missing data may introduce risk of bias and limit the interpretation of subgroup analyses. However, the overall methodological quality of the included studies was considered moderate-to-high.

### 3.8. Meta-Analysis and Publication Bias by Imaging Modality

#### 3.8.1. Perfusion-Based Methods

##### CT Perfusion (CTP)

**Figure 3:**
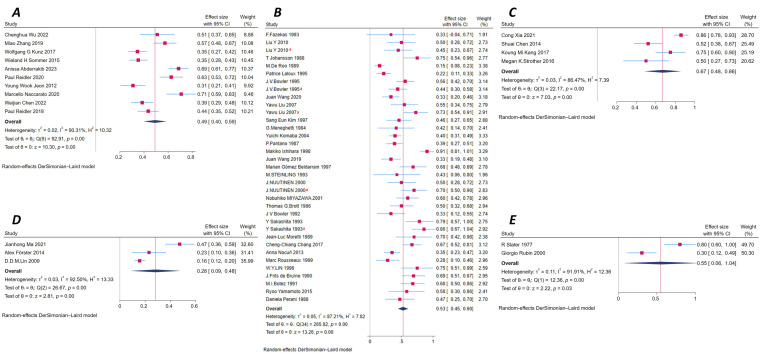
Forest plot of Perfusion-based methods including the following (**A**) CTP. (**B**) SPECT. (**C**) ASL MRI. (**D**) DSC-PWI. (**E**) by XeCT. ^a^ Indicates that the study reported multiple assessments of diaschisis within this subgroup.

Ten studies assessed diaschisis using CT perfusion imaging [14,38,39,40,41,42,43,44,45,46]. The pooled prevalence was 49% (95% CI: 40–59%). Heterogeneity among studies was high (I^2^ = 90.31%), indicating substantial variation, and thus the DerSimonian–Laird random-effects model was used. Egger’s regression test showed no significant evidence of publication bias (*p* = 0.36), and the funnel plot appeared approximately symmetrical. In the Galbraith plot, all data points fell within the 95% confidence boundaries, although some deviated from the central axis, indicating moderate variability. These studies included a total of 1066 ischemic stroke patients, with 507 diagnosed with diaschisis. Based on the type of occluded vessels, nine studies [14,38,39,40,41,42,44,45,46] reported involvement of large vessels occlusion (LVO), including the internal carotid artery (ICA), middle cerebral artery (MCA), and anterior cerebral artery (ACA), with a pooled prevalence of diaschisis of 51% (95% CI: 41–61%). In contrast, one study [43] did not specify large-vessel occlusion, with the reported prevalence being 31% (95% CI: 21–41%). According to [37]’s stroke phase, nine studies [14,39,40,41,42,43,44,45,46] were conducted in the acute phase and one study had an unclear time window [38]. The pooled prevalence of diaschisis during the acute phase was 49% (95% CI: 39–59%). CTP showed a moderate detection rate for diaschisis, with high heterogeneity but a low risk of publication bias (Supplementary Table S1, Figure 3A, and Supplementary Material S3A,a).

##### Single-Photon Emission Computed Tomography (SPECT)

A total of 30 studies (providing 35 datasets) used SPECT, making it the most extensively studied modality [7,12,15,25,26,27,29,47,48,49,50,51,52,53,54,55,56,57,58,59,60,61,62,63,64,65,66,67,68,69]. The isotopic tracer involved mainly including ^123^I-IMP (-HIPDM), ^99m^Tc-ECD (HMPAO), ^201^TI-DDC, and ^133^Xe. The pooled prevalence was 53% (95% CI: 45–60%), with high heterogeneity (I^2^ = 87.21%). A random-effects model was used for analysis. Egger’s test indicated no significant publication bias (*p* = 0.30). The funnel plot showed general symmetry, and the Galbraith plot suggested acceptable consistency, although a few studies deviated from the regression line. The included studies involved 959 patients, with 432 positive for diaschisis. According to the type of occluded vessels, 16 studies [7,12,15,25,27,29,51,52,56,57,58,59,61,63,64,68] (providing 20 datasets) reported the involvement of LVO, including the ICA, MCA, and ACA, with a pooled prevalence of diaschisis being 55% (95% CI: 45–64%). In total, 14 studies [26,47,48,49,50,53,54,55,60,62,65,66,67,69] (providing 15 datasets) did not specify large vessel occlusion with the reported prevalence was 50% (95% CI: 37–63%). Regarding stroke phase, 11 studies [25,26,27,29,47,50,51,55,58,59,69] (providing 15 datasets) were conducted in the acute phase, and pooled prevalence was 50% (95% CI: 37–62%); 11 studies [7,49,53,56,57,60,61,62,63,65,67] (providing 12 datasets) were conducted in the subacute and chronic phase, and pooled prevalence was 58% (95% CI: 46–69%). In total, 8 studies [12,15,48,52,54,64,66,68] had no clear time window. SPECT demonstrated good detection ability with moderate-to-high heterogeneity and low publication bias (Supplementary Table S1, Figure 3B, and Supplementary Material S3B,b).

##### Arterial Spin Labeling MRI (ASL MRI)

Four studies [9,16,70,71] employed ASL MRI, yielding a pooled prevalence of 67% (95% CI: 48–86%), the highest detection rate among all modalities. Heterogeneity was moderate (I^2^ = 86.47%). Due to the limited number of studies, Egger’s test was not performed. The Galbraith plot showed moderate dispersion, but within an acceptable range. The studies involved 181 patients, with 130 diagnosed with diaschisis. Based on the type of occluded vessels, one study [16] reported LVO with prevalence of diaschisis was 86% (95% CI: 78–93%). Three studies [9,70,71] did not mention large vessel occlusion, with the reported prevalence being 60% (95% CI: 43–77%), lower than patients who have LVO. Regarding stroke phase, one study [70] was conducted in the acute phase; prevalence was 75% (95% CI: 60–90%). Two studies [9,71] were conducted in the subacute and chronic phase, and the prevalence was 52% (95% CI: 39–64%). One study [16] had no clear time window. ASL MRI showed the highest detection rate (Supplementary Table S1, Figure 3C, and Supplementary Material S3c).

##### Dynamic Susceptibility Contrast Perfusion Imaging (DSC-PWI)

Three studies [11,17,72] used DSC-PWI, with a pooled prevalence of 28% (95% CI: 9–48%), the lowest among all modalities. Heterogeneity was extremely high (I^2^ = 92.50%), and a random-effects model was used. Galbraith plots showed significant deviations from the regression line, reflecting notable inconsistencies among studies.

A total of 414 patients were included, with 91 cases of diaschisis. All 3 studies [11,17,72] reported no-LVO and acute phase, with pooled prevalence of diaschisis being 28% (95% CI: 9–48%) (Supplementary Table S1, Figure 3D, and Supplementary Material S3d).

##### Xenon-Enhanced CT (XeCT)

Only two studies utilized XeCT [73,74]. The pooled prevalence was 55% (95% CI: 6–104%), with very high heterogeneity (I^2^ = 91.91%). The confidence interval was excessively wide, suggesting unstable results. Publication bias was not assessed due to the small number of studies. The studies involved 38 patients, with 19 showing diaschisis. These two studies [73,74] investigated LVO in the acute phase (Supplementary Table S1 and Figure S3E).

#### 3.8.2. Metabolism-Based and Functional Imaging Methods

##### Positron Emission Tomography (PET)

**Figure 4:**
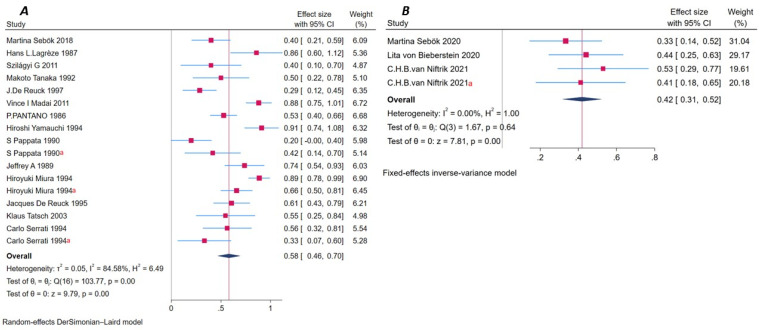
Forest plot for stroke patients assessed by Metabolism-based functional imaging: (**A**) PET. (**B**) fMRI. ^a^ Indicates that the study reported multiple assessments of diaschisis within this subgroup.

Seventeen datasets were extracted from 14 studies using PET imaging [5,6,8,18,28,30,75,76,77,78,79,80,81,82]. The isotopic tracer involved mainly including ^15^O and its compounds, ^18^FDG/^18^FCH_3_. The pooled prevalence of diaschisis was 58% (95% CI: 46–70%), which was among the highest across modalities. Among them, nine studies including eleven datasets [18,28,30,75,77,78,79,80,81] used ^15^O and its compounds as an isotopic tracer, and the overall prevalence was 61% (95% CI: 47–75%); two studies including three datasets [6,8] used both ^15^O with its compounds and ^18^FDG as an isotopic tracer, and the pooled prevalence was 29% (95% CI: 15–42%); and three studies [5,76,82] used ^18^FCH_3_ or ^18^FDG as a tracking agent, and the pooled prevalence was 73% (95% CI: 57–88%). Heterogeneity was substantial (I^2^ = 84.58%), and a random-effects model was applied. Egger’s test revealed no significant publication bias (*p* = 0.08), and the funnel plot showed acceptable symmetry. However, the Galbraith plot displayed several data points far from the central axis, suggesting a variation in effect sizes. A total of 297 stroke patients were included, with 177 diagnosed with diaschisis. According to the type of occluded vessels, ten studies [8,18,28,30,75,76,78,79,80,81] (providing 12 datasets) reported the involvement of LVO, including the ICA, MCA, and ACA, with a pooled prevalence of diaschisis of 62% (95% CI: 49–75%). Four studies [5,6,77,82] (providing five datasets) did not specify large vessel occlusion with the reported prevalence was 46% (95% CI: 25–67%). Regarding stroke phase, 1 studies [30] (providing two datasets) were conducted in acute phase, prevalence was 46% (95% CI: 23–68%). Ten studies [5,6,8,18,76,77,78,79,80,82] (providing 11 datasets) were conducted in the subacute and chronic phase; pooled prevalence was 53% (95% CI: 38–68%). Three studies [28,75,81] (providing four datasets) had no clear time window. PET exhibited a high detection rate and reasonable consistency (Supplementary Table S1, Figure 4A, and Supplementary Material S3C,e).

##### Functional MRI (fMRI)

Four studies (three articles) reported on fMRI-based detection of diaschisis [19,83,84]. The pooled prevalence was 42% (95% CI: 31–52%), with no observed heterogeneity (I^2^ = 0.00%). A fixed-effect model was used. Galbraith plots showed tight clustering along the central axis. Due to the small number of studies, publication bias was not assessed. A total of 66 patients were included, with 26 positive for diaschisis. Based on the type of occluded vessels, two studies [19,84] (three datasets) reported LVO with prevalence of diaschisis was 46% (95% CI: 33–59%). One study [83] did not mention large vessel occlusion with the reported prevalence was 33% (95% CI: 14–52%). According to stroke phase, all studies [19,83,84] were conducted in the subacute and chronic phase with the prevalence was 42% (95% CI: 31–52%). fMRI showed a moderate detection rate with excellent consistency (Supplementary Table S1, Figure 4B, and Supplementary Material S3f).

### 3.9. Diaschisis Subtypes: CCD, ITD, and Other Types

#### 3.9.1. Crossed Cerebellar Diaschisis (CCD)

**Figure 5:**
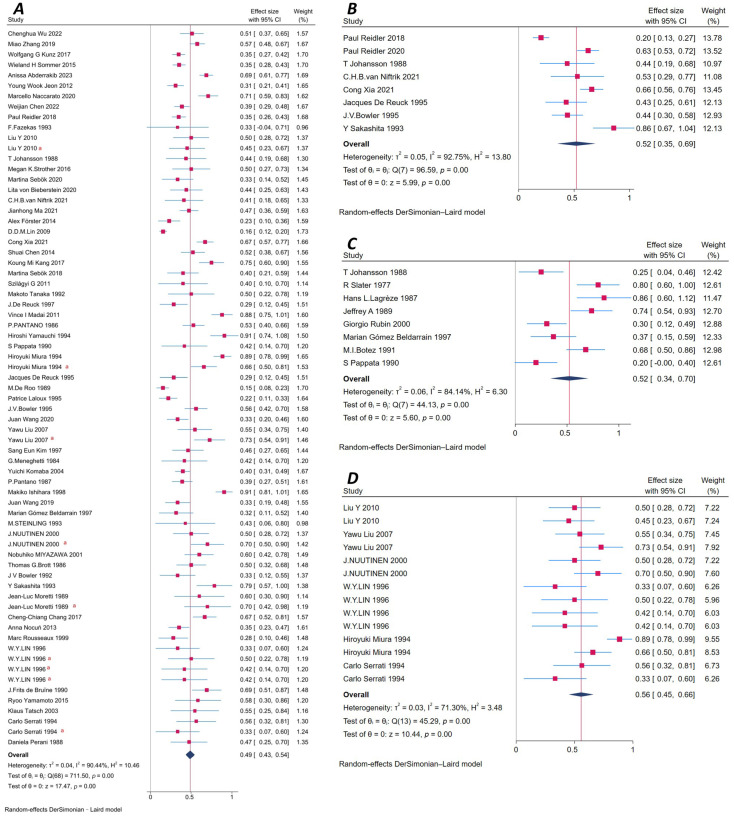
(**A**) Forest plot of crossed cerebellar diaschisis (CCD) in patients with ischemic stroke. (**B**) Forest plot of Ipsilateral Thalamic Diaschisis (ITD) in patients with ischemic stroke. (**C**) Forest plot of other Types of Diaschisis in patients with ischemic stroke. (**D**) Forest plot of diaschisis detection during the follow-up period after stroke. ^a^ Indicates that the study reported multiple assessments of diaschisis within this subgroup.

A total of 61 studies reported on CCD (Supplementary Table S1). The pooled prevalence was 49% (95% CI: 43–54%), with high heterogeneity (I^2^ = 90.44%). The DerSimonian-Laird random-effects model was used. Egger’s test showed no significant publication bias (*p* = 0.83), and the funnel plot was symmetric. The Galbraith plot showed that most studies were within the confidence boundaries, although some deviated from the axis, indicating acceptable consistency. Among 2833 patients, 1264 were identified with CCD. Six studies [25,26,27,28,29,30] performed follow-up imaging at various time points, including 3 days, 8 days, 1–3 weeks, and 5 years. These factors may influence the observed variability in CCD detection (Figure 5A and Supplementary Material S3D,g).

#### 3.9.2. Ipsilateral Thalamic Diaschisis (ITD)

Eight studies [16,19,42,46,49,56,58,79] evaluated ITD, with a pooled prevalence of 52% (95% CI: 35–69%). Heterogeneity was high (I^2^ = 92.75%), and a random-effects model was used. Egger’s test showed no evidence of publication bias (*p* = 0.68). The funnel plot was symmetric, and the Galbraith plot showed moderate variability. These studies included 433 stroke patients, of whom 205 had ITD (Figure 5B and Supplementary Material S3E,h).

#### 3.9.3. Other Types of Diaschisis

Eight studies [5,6,49,53,60,73,74,76] reported non-CCD, non-ITD types of diaschisis, including transhemispheric diaschisis, cerebello-cerebral diaschisis, ipsilateral cerebellar diaschisis, and ipsilateral cortical diaschisis. The pooled prevalence was 52% (95% CI: 34–70%) with high heterogeneity (I^2^ = 84.14%). Egger’s test showed no publication bias (*p* = 0.49), and the funnel plot was symmetric. The Galbraith plot showed notable deviations, indicating substantial variability across studies. These studies involved 139 patients, of whom 70 were diagnosed with other types of diaschisis (Figure 5C and Supplementary Material S3F,i).

### 3.10. Study Design and Follow-up Heterogeneity

Preliminary observations suggested that diaschisis detection rates might vary across different study designs (e.g., cross-sectional, retrospective, prospective) and geographic regions. Six included studies [25,26,27,28,29,30] reported diaschisis detection during follow-up after stroke. However, due to the wide variability in follow-up duration (from 3 days to 5 years) and inconsistent timing of assessments, these data were only presented descriptively (Figure 5D) and were not included in the pooled meta-analysis.

### 3.11. Influencing Factors Identified by Data-Driven Subgroup Analysis

Results are summarized in Table 1.

The subgroup analysis showed that age had a marginal effect on diaschisis detection, with a pooled SMD of 0.56 (95% CI: 0.47–0.64) in younger populations and 0.51 (95% CI: 0.44–0.57) in older populations. Exploratory subgroup analyses suggested that higher stroke severity (NIHSS ≥ 8.8) was associated with a modestly higher effect size for diaschisis detection compared with milder strokes. Study quality also had a modest impact: lower-quality studies (NOS < 7) showed slightly higher effect sizes (SMD = 0.55) than higher-quality studies (NOS ≥ 7; SMD = 0.50), suggesting possible overestimation in less rigorous studies. Regarding diaschisis type, studies on non-crossed cerebellar diaschisis yielded a higher pooled effect size (SMD = 0.66) compared with those focusing on crossed cerebellar diaschisis (SMD = 0.51). Detection varied substantially by imaging modality, with ASL-MRI (SMD = 0.67) and PET (SMD = 0.61) showing the highest effect sizes, followed by XeCT (0.55), SPECT (0.51), CTP (0.49), fMRI (0.39), and DSC-PWI (0.28), reflecting the higher physiological sensitivity of ASL and PET and the limited perfusion sensitivity of DSC-PWI. These findings should be interpreted cautiously, as subgroup thresholds were data-informed and intended to explore heterogeneity rather than define clinically prescriptive categories.

## 4. Discussion

This meta-analysis synthesized 66 studies including 3021 patients to provide a comprehensive estimate of diaschisis prevalence and to identify the main factors influencing its detection after ischemic stroke. Rather than comparing imaging techniques in a competitive sense, our goal was to determine whether diverse modalities converge on the same physiological phenomenon—namely, a remote reduction in perfusion, metabolism, or neural activity in structurally intact regions that are anatomically connected to the infarct. From this transmodal perspective, we found that diaschisis is frequent, with an overall pooled prevalence of 53% (95% CI: 47–58%). Crossed Cerebellar Diaschisis was the most commonly examined subtypes, yet thalamic and other remote forms showed comparable or even higher effect sizes, underscoring that diaschisis reflects network topology rather than any single favored pattern. Clinical modifiers such as stroke severity only had a modest impact on detection, and demographic factors such as age showed no measurable influence, suggesting that diaschisis is governed primarily by neuroanatomical disconnection. In contrast, the imaging modality exerted a strong effect: ASL-MRI and PET showed the highest pooled detection rates, whereas DSC-PWI detected diaschisis far less frequently. These differences likely reflect the specific physiological processes each technique measures and their sensitivity to subtle remote hypoperfusion or hypometabolism. Overall, our findings highlight diaschisis to be a robust and anatomically grounded consequence of focal stroke, detectable across modalities but variably expressed depending on both imaging physiology and lesion characteristics.

It is important to emphasize conceptual clarity. Diaschisis, as used here, refers to a cerebral phenomenon characterized by (i) a focal lesion, (ii) reduced activity/metabolism/perfusion in a remote but anatomically connected region, and (iii) a dynamic temporal course that may evolve all the time [2,85]. This formulation stresses diaschisis as a network-level consequence of focal injury—a functional disconnection—rather than a primary vascular lesion into the remote region. For this reason, we excluded studies that only assessed connectivity correlations without direct measures of perfusion or metabolism. That methodological distinction explains part of the observed modality-dependent variability and justifies our transmodal synthesis approach. The high between-study heterogeneity observed across analyses should not be viewed solely as a statistical limitation, but rather as a central finding of this meta-analysis. Rather than reflecting random noise, variability in reported diaschisis prevalence appears to be strongly and systematically driven by imaging methodology, highlighting the fundamentally physiological nature of the phenomenon. That methodological distinction explains part of the observed modality-dependent variability and justifies our transmodal synthesis approach. Several methodological and biological factors likely contribute to this heterogeneity. First, imaging thresholds and operational definitions of diaschisis varied markedly across studies, directly influencing reported prevalence. Second, timing is critical: imaging modalities differ in their typical window of use, and diaschisis itself may evolve dynamically over time. Third, patient selection and stroke characteristics—including lesion size, cortical versus subcortical location, and reperfusion status—strongly influence the likelihood of remote functional depression. Taken together, these sources of heterogeneity indicate that pooled prevalence estimates should be interpreted as an average across diverse methods and populations, rather than as a single “true” frequency applicable to any specific clinical context

A further important issue concerns the anatomical specificity of remote effects. Crossed cerebellar diaschisis (CCD) [8,9,17], involving disruption between supratentorial cortex and the contralateral cerebellum—regions outside a shared vascular territory—was the most commonly reported subtype, with a pooled prevalence of 49%. This reflects its ease of detection due to well-defined anatomical connections. The predominance of CCD in the literature reflects historical and methodological bias rather than exclusivity of the phenomenon, and non-cerebellar forms of diaschisis are likely underrepresented rather than absent. Less frequently studied variants, including ipsilateral thalamic diaschisis (ITD) and other forms (e.g., transhemispheric or cerebello-cerebral), were less represented in the literature but showed a similar pooled prevalence (52%), suggesting that disconnection patterns, rather than anatomical subtype alone, drive detection. These findings reinforce that diaschisis reflects a functional disconnection, not restricted by vascular boundaries, and that its detection depends heavily on imaging modality, stroke location, and clinical severity. Overall, our findings suggest that the larger and more strategically located the stroke, the more likely it is to induce remote functional disconnection, a process that may persist over time in some patients, based on descriptive longitudinal observations and is independent of patients’ age. The key clinical question remains whether such persistent diaschisis negatively affects long-term recovery. Future studies should investigate its prognostic value and potential reversibility. In particular, interventional approaches such as cerebellar neuromodulation [86] warrant exploration, as preliminary work has shown that modulating cerebellar excitability may help restore cortico-cerebellar communication and improve outcome [87,88].

Several potential factors may account for the observed variation in detection rates. These include differences in imaging mechanisms, timing of assessment, technological maturity, and patient characteristics. The relative strengths and limitations are intuitive and deserve careful appreciation rather than simplistic ranking. For instance, ASL-MRI is non-invasive, repeatable, and quantitative, qualities that increase sensitivity to persistent low-flow states in perfusion, but ASL protocols are not yet fully harmonized across centers [89,90]. In contrast, DSC-PWI relies on dynamic contrast-enhanced imaging, which may be less reliable in chronic vascular states [91]. Additionally, ASL is a relatively recent technique with more standardized protocols and advanced image post-processing, severe strokes (e.g., those with higher NIHSS scores or large vessel occlusion) may increase the likelihood of diaschisis, although such clinical details were inconsistently reported across studies [15,92]. Each imaging modality included in this review provides distinct physiological or pathophysiological information relevant to the detection of diaschisis.

Perfusion-based imaging techniques showed varying capacities for detecting diaschisis. CT Perfusion (CTP) [93,94,95] was exclusively used in the acute phase and proved useful for identifying early hemodynamic disturbances, but it showed only moderate sensitivity for diaschisis (SMD = 0.49), likely due to its limited spatial coverage and reduced ability to detect remote or subtle hypoperfusion in structurally intact areas. SPECT, also perfusion-based, had higher detection rates in subacute and chronic phases (58% vs. 50%) and was especially sensitive to crossed cerebellar diaschisis [96]. It effectively captured perfusion deficits in intact regions, supporting the concept of diaschisis as functional disconnection. Despite limitations in spatial resolution and quantification, SPECT remains clinically useful. In our meta-analysis, isotopic tracers such as ^99m^Tc-ECD (or HMPAO) and ^123^I-IMP (or HIPDM) serve as indirect measures of neuronal activity [97]. ASL-MRI had the highest pooled effect size (SMD = 0.67), reflecting its sensitivity to low-flow states and its noninvasive, repeatable nature [98]. These features make it particularly suited for subacute and chronic assessments, although technical variability and limited availability reduce its routine use [99,100,101]. DSC-PWI showed the lowest sensitivity (SMD = 0.28), likely because perfusion may appear preserved in deafferented regions with intact vasculature but suppressed metabolism, limiting its value for detecting diaschisis [11,17,72,102]. XeCT, used in older studies, demonstrated some sensitivity, but has fallen out of use due to technical and practical constraints [103].

Metabolism-based and functional imaging methods demonstrated stronger performance in detecting remote functional suppression. PET [104,105,106], used predominantly in non-acute phases, showed high sensitivity (SMD = 0.61) and captured persistent hypometabolism in structurally preserved but deafferented regions, supporting the view of diaschisis as synaptic downregulation. In our meta-analysis, the pooled prevalence in PET was 58%. The ^15^O and its compounds isotopic tracer in PET have the prevalence was 61%, although there are 2 studies with three datasets, both ^15^O with its compounds and ^18^FDG as isotopic tracer achieved a prevalence of 29%. However, studies using ^18^FCH or ^18^FDG as tracking agent with the prevalence was 73%. In PET studies, the prevalence of diaschisis varies significantly depending on the tracer used. PET remains the gold standard for metabolic assessment, although its clinical use is limited by high cost, radiation exposure, and restricted availability. fMRI, while not a direct metabolic measure, detected diaschisis through reduced resting-state connectivity and impaired interregional neural dynamics, particularly in cortico-cerebellar and thalamocortical circuits. Although only a small number of fMRI studies were included, all of them relied on BOLD-based measurements. While BOLD imaging does not directly quantify perfusion or metabolism, several of these studies reported reduced BOLD signal in remote, structurally preserved regions, consistent with functional depression, and therefore aligned with the physiological definition of diaschisis. Pure connectivity-based analyses were excluded. Because the objective of this meta-analysis was to estimate the prevalence of diaschisis across modalities—not to compare physiological mechanisms—BOLD fMRI data were retained when they demonstrated remote activity suppression. Their limited number also means that their contribution to pooled prevalence estimates was minimal, without affecting overall conclusions. Although not widely used clinically, fMRI offers valuable insights into network-level dysfunction, especially in chronic stroke [107,108,109]. Across imaging modalities, ASL-MRI and PET tended to yield higher prevalence estimates of diaschisis, particularly outside the acute phase, suggesting greater sensitivity to persistent network-level dysfunction. This likely reflects their ability to detect sustained reductions in cerebral blood flow or glucose metabolism in structurally intact but functionally deafferented regions. In such contexts, vascular supply may be preserved, while neuronal activity and metabolic demand are chronically suppressed, a pattern well captured by ASL and PET. In contrast, acute perfusion techniques such as CT perfusion and DSC-PWI, while highly valuable for infarct characterization and penumbral assessment, appear less suited to detecting subtle or remote functional impairment, particularly when diaschisis manifests as low-amplitude or chronic physiological depression rather than overt hemodynamic compromise. In contrast, CT perfusion and DSC-PWI are primarily designed to characterize acute hemodynamic compromise within infarcted or peri-infarct tissue. Their reliance on bolus contrast dynamics and thresholds optimized for ischemic core and penumbra may limit sensitivity to subtle, remote hypoperfusion occurring outside the primary vascular territory. As a result, these techniques may systematically underestimate diaschisis when it manifests as low-amplitude or chronic functional depression rather than frank flow limitation. Taken together, these modality-dependent differences suggest that diaschisis is not a unitary signal but a distributed physiological process that can manifest as altered perfusion, metabolism, or neural activity depending on disease stage and measurement technique. The observed heterogeneity therefore reinforces the concept of diaschisis as a network-level consequence of focal injury, rather than a binary or modality-specific entity.

From a clinical perspective, the relevance of diaschisis remains an open but important question. Exploratory analyses suggested that stroke severity, indexed by baseline NIHSS, may influence the likelihood of detecting diaschisis, whereas age showed a minor effect. Although the magnitude of the NIHSS-related effect was modest, this association is biologically plausible: more severe strokes are typically larger, involve more strategically connected cortical or subcortical hubs, and are therefore more likely to induce widespread network disruption and remote functional depression. In this context, diaschisis may index the extent of whole-network involvement, and thus partially contribute to worse early neurological deficits. However, given the exploratory, data-driven nature of these subgroup analyses and the incomplete reporting of NIHSS across studies, these findings should be viewed as hypothesis-generating rather than confirmatory. Whether diaschisis independently predicts long-term outcome beyond established clinical predictors such as age, baseline NIHSS, infarct volume, and reperfusion status requires dedicated prognostic studies. If confirmed, diaschisis could represent not only a marker of network vulnerability but also a potential therapeutic target. Early mechanistic and pilot interventional studies are promising and warrant further randomized evaluation to determine whether modulation of remote network excitability can reduce diaschisis and improve recovery.

### 4.1. Limitations

We must acknowledge some limitations of this study. First, diaschisis in this study was defined as reduced activity, metabolism, or perfusion in a remote but anatomically connected and structurally intact region following focal ischemic injury. Given the historical use of heterogeneous imaging modalities and thresholds, pooled prevalence estimates reflect convergence across diverse operational definitions rather than a single uniform criterion, contributing to observed heterogeneity. Second, the substantial between-study heterogeneity and inconsistent reporting (some studies omitted key variables such as precise time from stroke to imaging, reperfusion status, NIHSS scores at admission, occluded vessel types, or other clinical characteristics) limited subgroup and meta-regression analyses. In the presence of substantial heterogeneity, pooled prevalence estimates should not be interpreted as a precise value applicable to individual patients or specific clinical settings; rather, they represent an average probability of detecting diaschisis across diverse imaging modalities, populations, and time points. Importantly, the consistency of moderate-to-high prevalence across modalities supports the robustness of diaschisis as a network-level phenomenon, despite variability in its measured expression. The exclusion of purely connectivity-based fMRI work narrows the scope to perfusion/metabolic phenomena and may miss cases where functional disconnection is present without measurable hemodynamic changes, but this choice was deliberate to preserve conceptual coherence. Third, the classification of study designs (e.g., cross-sectional, retrospective, prospective) was unclear in some studies, and the uneven distribution of studies across countries and regions raised the possibility of misclassification and geographic bias. Fourth, given the limited number of studies and relatively small sample sizes in ASL-MRI and XeCT, the estimates for those techniques should be interpreted cautiously and viewed as exploratory rather than definitive. Finally, longitudinal data are sparse and variable in timing; because diaschisis may be transient in some patients and persistent in others, cross-sectional prevalence estimates cannot characterize the temporal trajectory of diaschisis, which may be transient in some patients and persistent in others.

### 4.2. Clinical Relevance, Imaging Strategy, and Perspectives

This meta-analysis showed that diaschisis appears to be a common, often persistent, network-level consequence of focal ischemic injury, and highlights the high prevalence (53%). Its detection is modality- and time-dependent and is more likely in patients with more severe strokes, particularly crossed cerebellar diaschisis (CCD), with detection rates increasing with stroke severity and remaining relatively stable over time.

These findings open several avenues for future research. First, voxel-based lesion–perfusion mapping in large stroke cohorts may help identify which lesion locations are most consistently associated with remote perfusion deficits, improving our mechanistic understanding of functional disconnection beyond classical vascular territories or stroke volume. Second, although both FDG-PET and ASL-MRI demonstrated the highest sensitivity for detecting diaschisis (SMD = 0.61 and 0.67, respectively), their comparative performance warrants further exploration. ASL-MRI offers significant advantages for routine clinical use and longitudinal follow-up due to its non-invasive, radiation-free nature, while FDG-PET remains the gold standard for assessing metabolic suppression. Emerging hybrid PET-MRI systems may help reconcile these approaches by enabling simultaneous evaluation of perfusion and metabolism within a single session, facilitating a more comprehensive characterization of diaschisis [3,110]. Finally, the clinical relevance of diaschisis remains to be established. Future studies should investigate whether the presence of diaschisis independently contributes to poorer long-term outcomes and whether it represents a modifiable target through interventions such as non-invasive brain stimulation aimed at restoring functional connectivity in remote but viable brain regions. Only with such harmonized, mechanistic studies can we determine whether diaschisis is a marker of irreversible deficit or a modifiable mediator of recovery- and hence whether it should be a target for therapeutic modulation.

## 5. Conclusions

In conclusion, diaschisis emerges as a frequent and robust network-level consequence of ischemic stroke. While lesion topology and anatomical disconnection constitute the fundamental substrate of remote functional depression, our findings indicate that diaschisis detection reflects an interaction between lesion anatomy, clinical severity, and the physiological properties of the imaging modality used. Clinical factors such as stroke severity appear to play a modest but biologically plausible modulatory role, whereas demographic factors such as age exert little influence. The marked modality dependence of detection underscores that diaschisis is not a unitary signal, but rather a physiological phenomenon variably expressed across perfusion, metabolic, and functional imaging techniques. These results highlight the need for modality-aware interpretation, standardized definitions, and longitudinal multimodal protocols to clarify the temporal evolution and clinical significance of diaschisis.

## Figures and Tables

**Table 1:** Subgroup analysis of potential factors influencing the detection rate of diaschisis.

Sub-Analysis	SMD	95%CI	Diaschisis	Total	I^2^	*p*
Age < 63 y	0.56	0.47, 0.64	517	928	87.83%	<0.001
Age ≥ 63 y	0.51	0.44, 0.57	700	1487	84.46%	<0.001
NIHSS < 8.8	0.52	0.33, 0.72	172	304	93.08%	<0.001
NHISS ≥ 8.8	0.55	0.43, 0.67	382	744	91.96%	<0.001
NOS < 7	0.55	0.44, 0.66	427	1043	93.72%	<0.001
NOS ≥ 7	0.50	0.43, 0.56	955	1978	89.50%	<0.001
CCD	0.51	0.44, 0.57	1264	2833	92.65%	<0.001
No CCD	0.66	0.52, 0.80	118	188	73.00%	<0.001
CTP	0.49	0.40, 0.59	507	1066	90.31%	<0.001
SPECT	0.51	0.42, 0.59	432	959	87.64%	<0.001
ASL MRI	0.67	0.48, 0.86	130	181	86.47%	<0.001
DSC-PWI	0.28	0.09, 0.48	91	414	92.50%	0.005
XeCT	0.55	0.06, 1.04	19	38	91.91%	0.026
PET	0.61	0.49, 0.74	177	297	84.32%	<0.001
fMRI	0.39	0.28, 0.51	26	66	0.00%	<0.001

*p* < 0.05 was considered statistically significant.

## Data Availability

No new data were created or analyzed in this study. Data sharing is not applicable to this article.

## Supplementary Materials

The following supporting information can be downloaded at https://www.mdpi.com/article/10.3390/brainsci16010050/s1. Supplementary Material S1: PRISMA 2020 checklist; Supplementary Material S2: Full strategy; Supplementary Material S3: A–F: A is funnel plot for ischemic stroke with CTP. B is funnel plot for ischemic stroke with SPECT. C is funnel plot for ischemic stroke with PET. D is funnel plot for crossed cerebellar diaschisis (CCD) in patients with ischemic stroke. E is funnel plot for Ipsilateral Thalamic Diaschisis (ITD) in patients with ischemic stroke. F is funnel plot for other Types of Diaschisis in patients with ischemic stroke. Table S1: Data extraction of clinical aspects of patients with ischemic stroke in seven neuroimaging modalities. Reference [111] is cited in the Supplementary Materials.
