# Supplementary material for: Prevalence and Imaging Correlates of Cerebral Diaschisis After Ischemic Stroke: A Systematic Review and Meta-Analysis
Source: Brain Sci. 2025 Dec 29;16(1):50. doi: 10.3390/brainsci16010050 (PMC12839182; doi:10.3390/brainsci16010050)
Supplement: Supplementary file 1 [file brainsci-16-00050-s001.zip › brainsci-4061369-supplementary.pdf]

Supplementary Material S1

Section and Topic	Item #	Checklist item	Location where item is reported
**TITLE**			
Title	1	Identify the report as a systematic review.	Title (pg1)
**ABSTRACT**			
Abstract	2	See the PRISMA 2020 for Abstracts checklist.	Abstract (pg1)
**INTRODUCTION**			
Rationale	3	Describe the rationale for the review in the context of existing knowledge.	Introduction (pg1-3), Figure 1
Objectives	4	Provide an explicit statement of the objective(s) or question(s) the review addresses.	Introduction (pg2)
**METHODS**			
Eligibility criteria	5	Specify the inclusion and exclusion criteria for the review and how studies were grouped for the syntheses.	Methods (pg3-4)
Information sources	6	Specify all databases, registers, websites, organisations, reference lists and other sources searched or consulted to identify studies. Specify the date when each source was last searched or consulted.	Methods (pg3-5), Figure 2
Search strategy	7	Present the full search strategies for all databases, registers and websites, including any filters and limits used.	Methods (pg3), Supplementary 2
Selection process	8	Specify the methods used to decide whether a study met the inclusion criteria of the review, including how many reviewers screened each record and each report retrieved, whether they worked independently, and if applicable, details of automation tools used in the process.	Methods (pg3-4), supplementary Table 1 and Figure 2
Data collection process	9	Specify the methods used to collect data from reports, including how many reviewers collected data from each report, whether they worked independently, any processes for obtaining or confirming data from study investigators, and if applicable, details of automation tools used in the process.	Methods (pg3-5), supplementary Table 1 and Figure 2
Data items	10a	List and define all outcomes for which data were sought. Specify whether all results that were compatible with each outcome domain in each study were sought (e.g. for all measures, time points, analyses), and if not, the methods used to decide which results to collect.	Methods (pg3-5)
	10b	List and define all other variables for which data were sought (e.g. participant and intervention characteristics, funding sources). Describe any assumptions made about any missing or unclear information.	Methods (pg3-6)
Study risk of bias assessment	11	Specify the methods used to assess risk of bias in the included studies, including details of the tool(s) used, how many reviewers assessed each study and whether they worked independently, and if applicable, details of automation tools used in the process.	Methods (pg3-4)
Effect measures	12	Specify for each outcome the effect measure(s) (e.g. risk ratio, mean difference) used in the synthesis or presentation of results.	Methods (pg3-5)
Synthesis methods	13a	Describe the processes used to decide which studies were eligible for each synthesis (e.g. tabulating the study intervention characteristics and comparing against the planned groups for each synthesis (item #5)).	Methods (pg3-5), Supplementary Table 1
	13b	Describe any methods required to prepare the data for presentation or synthesis, such as handling of missing summary statistics, or data conversions.	Methods (pg3-5), Supplementary Table 1
	13c	Describe any methods used to tabulate or visually display results of individual studies and syntheses.	Supplementary Table 1
	13d	Describe any methods used to synthesize results and provide a rationale for the choice(s). If meta-analysis was performed, describe the model(s), method(s) to identify the presence and extent of statistical heterogeneity, and software package(s) used.	Methods (pg3-6)
	13e	Describe any methods used to explore possible causes of heterogeneity among study results (e.g. subgroup analysis, meta-	Methods (pg3-6)

Section and Topic	Item #	Checklist item	Location where item is reported
		regression).	
	13f	Describe any sensitivity analyses conducted to assess robustness of the synthesized results.	Methods (pg3-6)
Reporting bias assessment	14	Describe any methods used to assess risk of bias due to missing results in a synthesis (arising from reporting biases).	Methods (pg3-6)
Certainty assessment	15	Describe any methods used to assess certainty (or confidence) in the body of evidence for an outcome.	Methods (pg4-6)
**RESULTS**			
Study selection	16a	Describe the results of the search and selection process, from the number of records identified in the search to the number of studies included in the review, ideally using a flow diagram.	Results (pg6-7), Figure 2
	16b	Cite studies that might appear to meet the inclusion criteria, but which were excluded, and explain why they were excluded.	Results (pg6-7), Figure 2
Study characteristics	17	Cite each included study and present its characteristics.	Results (pg6-7)
Risk of bias in studies	18	Present assessments of risk of bias for each included study.	Results(pg7-8)
Results of individual studies	19	For all outcomes, present, for each study: (a) summary statistics for each group (where appropriate) and (b) an effect estimate and its precision (e.g. confidence/credible interval), ideally using structured tables or plots.	Results(pg7-8), Supplementary Table 1
Results of syntheses	20a	For each synthesis, briefly summarise the characteristics and risk of bias among contributing studies.	Results(pg7-8), Supplementary 3
	20b	Present results of all statistical syntheses conducted. If meta-analysis was done, present for each the summary estimate and its precision (e.g. confidence/credible interval) and measures of statistical heterogeneity. If comparing groups, describe the direction of the effect.	Results(pg6-14),Figure3-5, Supplementary Table 1
	20c	Present results of all investigations of possible causes of heterogeneity among study results.	Results(pg6-14),Figure3-5, Supplementary 3
	20d	Present results of all sensitivity analyses conducted to assess the robustness of the synthesized results.	Results(pg6-14),Figure3-5, Supplementary 3
Reporting biases	21	Present assessments of risk of bias due to missing results (arising from reporting biases) for each synthesis assessed.	Results(pg6-14),Figure3-5, Table 1, Supplementary 3
Certainty of evidence	22	Present assessments of certainty (or confidence) in the body of evidence for each outcome assessed.	Results(pg6-14),Figure3-5, Table 1, Supplementary 3
**DISCUSSION**			
Discussion	23a	Provide a general interpretation of the results in the context of other evidence.	Discussion (pg14-17)
	23b	Discuss any limitations of the evidence included in the review.	Discussion (pg14-17)
	23c	Discuss any limitations of the review processes used.	Discussion (pg16)
	23d	Discuss implications of the results for practice, policy, and future research.	Discussion (pg16-17)
**OTHER INFORMATION**			
Registration and protocol	24a	Provide registration information for the review, including register name and registration number, or state that the review was not registered.	Methods (pg3)

Section and Topic	Item #	Checklist item	Location where item is reported
	24b	Indicate where the review protocol can be accessed, or state that a protocol was not prepared.	Methods (pg3-4)
	24c	Describe and explain any amendments to information provided at registration or in the protocol.	Methods (pg3-4)
Support	25	Describe sources of financial or non-financial support for the review, and the role of the funders or sponsors in the review.	Acknowledgments (pg17)
Competing interests	26	Declare any competing interests of review authors.	Conflict of Interest statement (pg17)
Availability of data, code and other materials	27	Report which of the following are publicly available and where they can be found: template data collection forms; data extracted from included studies; data used for all analyses; analytic code; any other materials used in the review.	Methods (pg 3-4)

From: Page MJ, McKenzie JE, Bossuyt PM, Boutron I, Hoffmann TC, Mulrow CD, et al. The PRISMA 2020 statement: an updated guideline for reporting systematic reviews. BMJ 2021;372:n71. doi: 10.1136/bmj.n71. This work is licensed under CC BY 4.0. To view a copy of this license, visit https://creativecommons.org/licenses/by/4.0/

Registered records published and stable

ID	Review	Date created	Last edited
CRD420251017909	Prevalence and Detection Rates of Diaschisis in Ischemic Stroke Patients Using Different Imaging Techniques: A Systematic Review and Meta-Analysis	23 March 2025	24 March 2025

Supplementary Material S2

PubMed (search before 2024.2.24)

1. The MeSH words for Stroke:

Stroke; Strokes; Cerebrovascular Accident; Cerebrovascular Accidents; CVA (Cerebrovascular Accident); CVAs (Cerebrovascular Accident); Cerebrovascular Apoplexy; Apoplexy, Cerebrovascular; Vascular Accident; Brain; Brain Vascular Accident; Brain Vascular Accidents; Vascular Accidents; Brain, Cerebrovascular Stroke; Cerebrovascular Strokes; Stroke, Cerebrovascular; Strokes, Cerebrovascular, Apoplexy; Cerebral Stroke; Cerebral Strokes; Stroke, Cerebral; Strokes, Cerebral; Stroke, Acute; Acute Stroke; Acute Strokes; Strokes, Acute; Cerebrovascular Accident, Acute; Acute Cerebrovascular Accident; Acute Cerebrovascular Accidents; Cerebrovascular Accidents, Acute

2. The MeSH words for Diaschisis/Functional disconnection:

Diaschisis; Connectional Diaschisis; Diaschisis, Connectional; Focal Diaschisis; Diaschisis, Focal; functional disconnection

3. The overall search strategy:

(((((((((((((((((((((((((((((Stroke) OR (Strokes)) OR (Cerebrovascular Accident)) OR (Cerebrovascular Accidents)) OR (CVA (Cerebrovascular Accident))) OR (CVAs (Cerebrovascular Accident))) OR (Cerebrovascular Apoplexy)) OR (Apoplexy, Cerebrovascular)) OR (Vascular Accident, Brain)) OR (Brain Vascular Accident)) OR (Brain Vascular Accidents)) OR (Vascular Accidents, Brain)) OR (Cerebrovascular Stroke)) OR (Cerebrovascular Strokes)) OR (Stroke, Cerebrovascular)) OR (Strokes, Cerebrovascular)) OR (Apoplexy)) OR (Cerebral Stroke)) OR (Cerebral Strokes)) OR (Stroke, Cerebral)) OR (Strokes, Cerebral)) OR (Stroke, Acute)) OR (Acute Stroke)) OR (Acute Strokes)) OR (Strokes, Acute)) OR (Cerebrovascular Accident, Acute)) OR (Acute Cerebrovascular Accident)) OR (Acute Cerebrovascular Accidents)) OR (Cerebrovascular Accidents, Acute)) AND ((((((Diaschisis) OR (Connectional Diaschisis)) OR (Diaschisis, Connectional)) OR (Focal Diaschisis)) OR (Diaschisis, Focal)) OR (functional disconnection))

Embase (search before 2024.2.24)

1. The MeSH words for Stroke：

'accident, cerebrovascular' OR 'acute cerebrovascular lesion' OR 'acute focal cerebral vasculopathy' OR 'acute stroke' OR 'apoplectic stroke' OR 'apoplexia' OR 'apoplexy' OR 'blood flow disturbance, brain' OR 'brain accident' OR 'brain attack' OR 'brain blood flow disturbance' OR 'brain insult' OR 'brain insultus' OR 'brain vascular accident' OR 'cerebral apoplexia' OR 'cerebral insult' OR 'cerebral stroke' OR 'cerebral vascular accident' OR 'cerebral vascular insufficiency' OR 'cerebro vascular accident' OR 'cerebrovascular arrest' OR 'cerebrovascular failure' OR 'cerebrovascular injury' OR 'cerebrovascular insufficiency' OR 'cerebrovascular insult' OR 'cerebrum vascular accident' OR 'cryptogenic stroke' OR 'CVA' OR 'insultus cerebralis' OR 'ischaemic seizure' OR 'ischemic seizure' OR 'stroke' OR 'thrombotic stroke' OR 'cerebrovascular accident'

2. The MeSH words for Diaschisis/Functional disconnection：

'diaschises' OR 'diaschisis' OR 'functional disconnection'

3. The overall search strategy:

('functional disconnection' OR 'diaschises'/exp OR 'diaschises' OR 'diaschisis'/exp OR 'diaschisis') AND ('accident, cerebrovascular'/exp OR 'accident, cerebrovascular' OR 'acute cerebrovascular lesion'/exp OR 'acute cerebrovascular lesion' OR 'acute focal cerebral vasculopathy'/exp OR 'acute focal cerebral vasculopathy' OR 'acute stroke'/exp OR 'acute stroke' OR 'apoplectic stroke'/exp OR 'apoplectic stroke' OR 'apoplexia'/exp OR 'apoplexia' OR 'apoplexy'/exp OR 'apoplexy' OR 'blood flow disturbance, brain'/exp OR 'blood flow disturbance, brain' OR 'brain accident'/exp OR 'brain accident' OR 'brain attack'/exp OR 'brain attack' OR 'brain blood flow disturbance'/exp OR 'brain blood flow disturbance' OR 'brain insult'/exp OR 'brain insult' OR 'brain insultus'/exp OR 'brain insultus' OR 'brain vascular accident'/exp OR 'brain vascular accident' OR 'cerebral apoplexia'/exp OR 'cerebral apoplexia' OR 'cerebral insult'/exp OR 'cerebral insult' OR 'cerebral stroke'/exp OR 'cerebral stroke' OR 'cerebral vascular accident'/exp OR 'cerebral vascular accident' OR 'cerebral vascular insufficiency'/exp OR 'cerebral vascular insufficiency' OR 'cerebro vascular accident'/exp OR 'cerebro vascular accident' OR 'cerebrovascular arrest'/exp OR 'cerebrovascular arrest' OR 'cerebrovascular failure'/exp OR 'cerebrovascular failure' OR 'cerebrovascular injury'/exp OR 'cerebrovascular injury' OR 'cerebrovascular insufficiency'/exp OR 'cerebrovascular insufficiency' OR 'cerebrovascular insult'/exp OR 'cerebrovascular insult' OR 'cerebrum vascular accident'/exp OR 'cerebrum vascular accident' OR 'cryptogenic stroke'/exp OR 'cryptogenic stroke' OR 'cva'/exp OR 'cva' OR 'insultus cerebralis'/exp OR 'insultus cerebralis' OR 'ischaemic seizure'/exp OR 'ischaemic seizure' OR 'ischemic seizure'/exp OR 'ischemic seizure' OR 'stroke'/exp OR 'stroke' OR 'thrombotic stroke'/exp OR 'thrombotic stroke' OR 'cerebrovascular accident'/exp OR 'cerebrovascular accident')

Supplementary Material S3

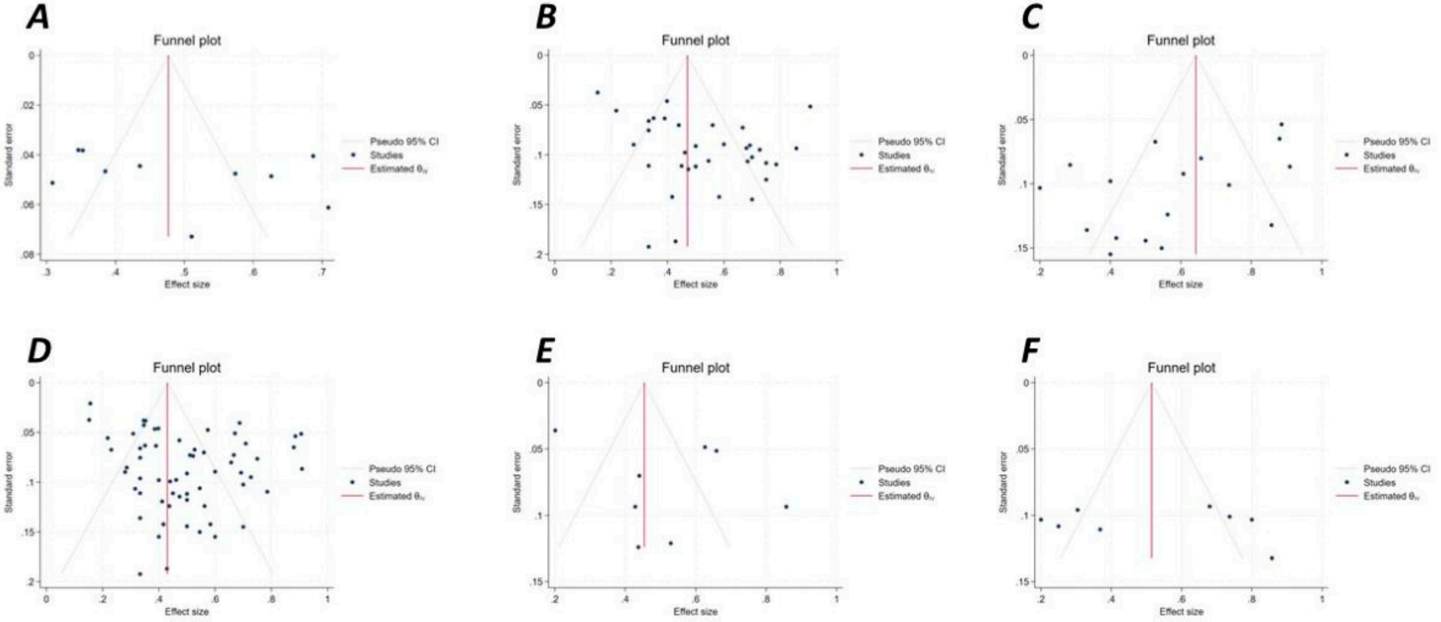

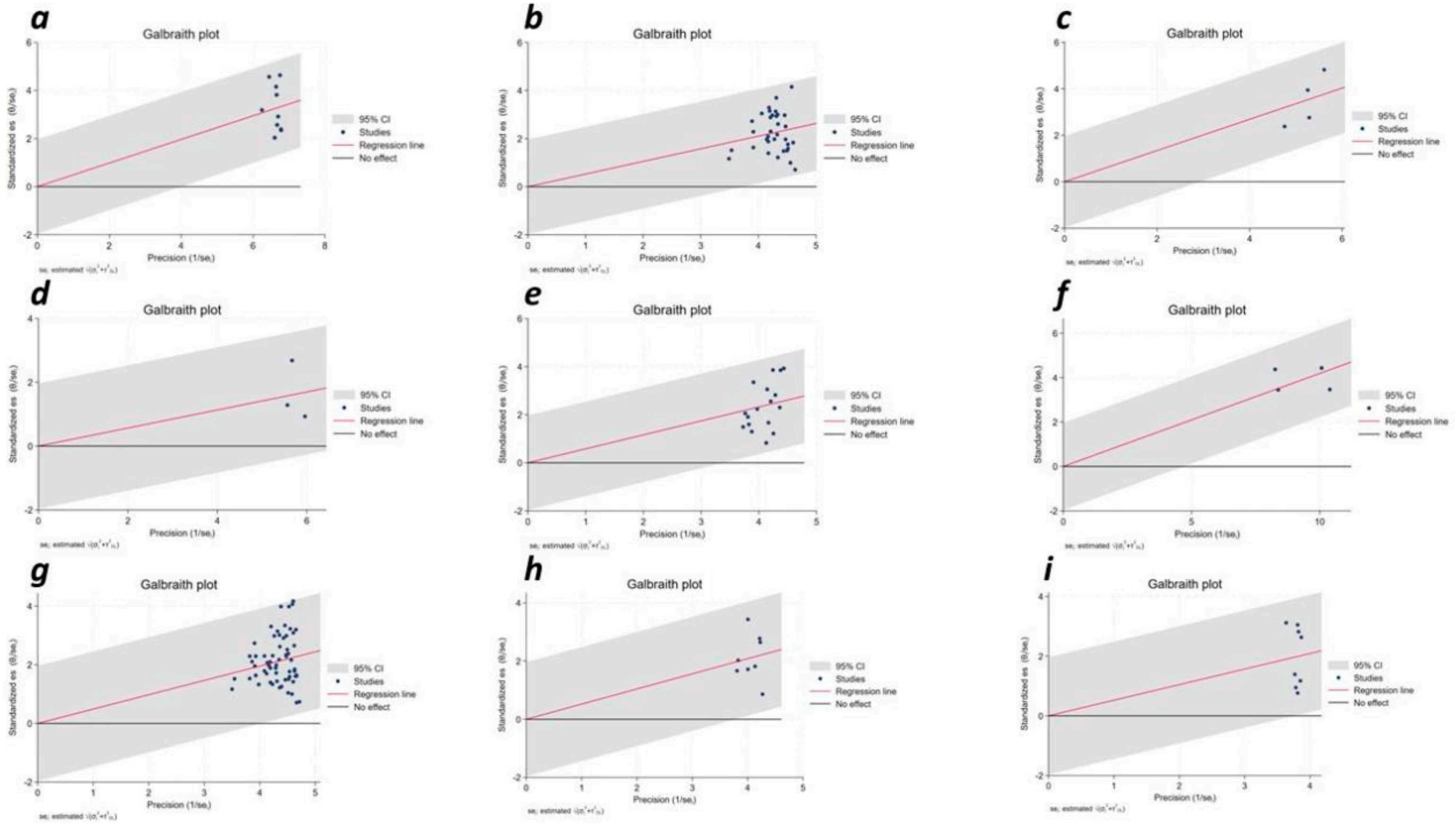

***Supplementary Material S3**: **A-F**: **A** is funnel plot for ischemic stroke with CTP. **B** is funnel plot for ischemic stroke with SPECT. **C** is funnel plot for ischemic stroke with PET. **D** is funnel plot for crossed cerebellar diaschisis (CCD) in patients with ischemic stroke. **E** is funnel plot for Ipsilateral Thalamic Diaschisis (ITD) in patients with ischemic stroke. **F** is funnel plot for other Types of Diaschisis in patients with ischemic stroke. **a-i**: **a** is Galbraith plot for ischemic stroke with CTP. **b** is Galbraith plot for ischemic stroke with SPECT. **c** is Galbraith plot for ischemic stroke with ASL MRI. **d** is Galbraith plot for ischemic stroke with DSC-PWI. **e** is Galbraith plot for ischemic stroke with PET. **f** is Galbraith plot for ischemic stroke with fMRI. **g** is Galbraith plot for crossed cerebellar diaschisis (CCD) in patients with ischemic stroke. **h** is Galbraith plot for Ipsilateral Thalamic Diaschisis (ITD) in patients with ischemic stroke. **i** is Galbraith plot for other Types of Diaschisis in patients with ischemic stroke.*

Supplementary Table S1.

Supplementary Table S1: Data extraction of clinical aspects of patients with ischemic stroke in seven neuroimaging modalities												
Reference	Patients and Gender	Age	Study type	Country and Continent	NOS	NHISS admission	After the onset of Stroke	Type of vessels	Type of Diaschisis	Follow-up	Isotopic tracer	Modality
Chenghua Wu 2022	n=47, M 28, F 19	62.49±8.16	Cross-sectional	China, Asia	8	NHISS admission: 2.46±2.95	After the onset of Stroke: 24h-3m	Type of vessels: ICA/MCA	CCD=24	No follow-up	Iohexol	CTP
Miao Zhang 2019	n=108, M 76, F 32	65.6±15.8	Retrospective cohort	China, Asia	7	No NHISS admission	After the onset of Stroke: ≤6h	Type of vessels: ICA/MCA/ACA	CCD=62	No follow-up	Iodinated contrast material	VPCT
Wolfgang G Kunz 2017	n=156, M 69, F 87	73(58-82)	Retrospective cohort	Germany, Europe	9	NHISS admission: 14(9-17)	After the onset of Stroke: ≤2d	Type of vessels: ICA/MCA	CCD=54	No follow-up	Iohexol	WB-CTP
Wieland H Sommer 2015	n=156, M 44, F 112	69.7±14.9	Case-control	Germany, Europe	7	No NHISS admission	After the onset of Stroke: ≤1d	Type of vessels: MCA	CCD=55	No follow-up	Iodinated contrast material	WB-CTP
Anissa Abderrakib 2023	n=131	70.6±14.8	Cross-sectional	Belgium, Europe	8	NHISS admission: 15.9±7.8	After the onset of Stroke: ≤7.5h	Type of vessels: ICA/MCA/ACA	CCD=90	No follow-up	Iodinated contrast material	CTP
Paul Reidler 2020	n=99, M 49, F 50	75(63-81)	Cross-sectional	Germany, Europe	9	NHISS admission: 14(9-17)	After the onset of Stroke: ≤24h	Type of vessels: ICA/MCA	ITD=62	No follow-up	Iodinated contrast material	CTP
Young Wook Jeon 2012	n=81	Unknown	Retrospective cohort	Korea, Asia	7	No NHISS admission	After the onset of Stroke: ≤24h	Type of vessels: Supratentorial	CCD=25	No follow-up	Iohexol	CTP
Marcello Naccarato 2020	n=55, M 25, F 30	72.3±15.6	Cross-sectional	Italy, Europe	7	NHISS admission: 6(1-22)	After the onset of Stroke: ≤4.5h	Type of vessels: MCA	CCD=39	No follow-up	Contrast medium	CTP
Weijian Chen 2022	n=109, M 61, F 48	68.7±12.9	Retrospective cohort	China, Asia	8	NHISS admission: 10(4-16)	After the onset of Stroke: ≤6h	Type of vessels: ICA/MCA/ACA	CCD=42	No follow-up	Nonionic iodine	CTP
Paul Reidler 2018	n=124, M 55, F 69	73(58-81)	Cross-sectional	Germany, Europe	8	NHISS admission: 14(9-16)	After the onset of Stroke: ＜5d	Type of vessels: MCA	CCD+ITD=54	No follow-up	Iodinated contrast material	CTP
F.Fazekas 1993	n=6, M 3, F 3	63.2±7.2	Cross-sectional	Austria, Europe	4	No NHISS admission	After the onset of Stroke: ＜1week	Type of vessels: Upper Pontine	CCD=2	No follow-up	^{123}I-IMP	SPECT
Liu Y 2010	n=20, M 12, F 8	70±9	Retrospective cohort	Finland, Europe	5	No NHISS admission	After the onset of Stroke: ≤48h	Type of vessels: ICA	CCD=10, CCD=9 (8d)	8 day Follow-up	^{99m}Tc-ECD	SPECT
T Johansson 1988	n=16, M 10, F 6	66(57-75)	Cross-sectional	Sweden, Europe	5	No NHISS	After the onset of Stroke: 2m	Type of vessels:	CCD+ITD+Transhemispheric Diaschisis=12	No follow-up	^{123}I-IMP	SPECT

						admission		cortical/central cerebral				
M.De Roo 1989	n=92	Unknown	Cross-sectional	Belgium, Europe	4	No NHISS admission	After the onset of Stroke: ≤1w	Type of vessels: Supratentorial	CCD=14	No follow-up	^{99m}Tc-HMPAO	SPECT
Patrice Laloux 1995	n=55, M 38, F 17	71±9	Prospective cohort	Belgium, Europe	7	No NHISS admission	After the onset of Stroke: ＜12h	Type of vessels: ICA	CCD=12	No follow-up	^{99m}Tc-HMPAO	SPECT
J.V.Bowler 1995	n=50, M 22, F 28	67.86±12.9	Cross-sectional	UK, Europe	6	No NHISS admission	After the onset of Stroke: 0.8d-1.7d	Type of vessels: ICA/MCA	CCD=28, ITD=22	No follow-up	^{99m}Tc-HMPAO	SPECT
Juan Wang 2020	n=51, M 35, F 16	62.16±12.16	Cross-sectional	China, Asia	7	NHISS admission: 3.12±2.16	After the onset of Stroke: 24h-2w	Type of vessels: MCA	CCD=17	No follow-up	^{99m}Tc-ECD	SPECT
Yawu Liu 2007	n=22, M 14, F 8	70±9	Retrospective cohort	Finland, Europe	8	NHISS admission: 9.09±7.2	After the onset of Stroke: ≤48h	Type of vessels: Supratentorial	CCD=12, CCD=16 (8d)	8d Follow-up	^{99m}Tc-ECD	SPECT
Sang Eun Kim 1997	n=26, M 18, F 8	55(34-77)	Cross-sectional	Korea, Asia	6	No NHISS admission	After the onset of Stroke: 5d-6.2y	Type of vessels: ICA	CCD=12	No follow-up	^{99m}Tc-HMPAO	SPECT
G.Meneghetti 1984	n=12, M 8, F 4	60.42±13.42	Cross-sectional	Denmark, Europe	6	No NHISS admission	After the onset of Stroke: ≤3d	Type of vessels: Supratentorial	CCD=5	No follow-up	^{133}Xe	SPECT
Yuichi Komaba 2004	n=113, M 75, F 38	66±13	Retrospective cohort	Japan, Asia	8	No NHISS admission	After the onset of Stroke: ≤1m	Type of vessels: ICA	CCD=45	No follow-up	^{123}I-IMP	SPECT
P.Pantano 1987	n=59, M 46, F 13	58±11.5	Cross-sectional	Italy, Europe	7	No NHISS admission	After the onset of Stroke: ≤5y	Type of vessels: ICA	CCD=23	No follow-up	^{123}I-HIPDM	SPECT
Makiko Ishihara 1998	n=32, M 17, F 15	61(20-87)	Retrospective cohort	Japan, Asia	6	No NHISS admission	After the onset of Stroke: ≤3m	Type of vessels: MCA	CCD=29	No follow-up	^{123}I-IMP	SPECT
Juan Wang 2019	n=39, M 25, F 14	62.41±12.97	Retrospective cohort	China, Asia	7	NHISS admission: 3.15±2.21	After the onset of Stroke: 24h-2w	Type of vessels: ICA	CCD=13	No follow-up	^{99m}Tc-ECD	SPECT
Marian Gómez Beldarrain 1997	n=19	Unknown	Case-control	Spain, Europe	5	No NHISS admission	After the onset of Stroke: ≥3w	Type of vessels: Cerebellar	CCD+ICD=13	No follow-up	^{99m}Tc-HMPAO	SPECT
M.STEINLING 1993	n=7, M 3, F 4	54(33-70)	Cross-sectional	France, Europe	5	No NHISS admission	After the onset of Stroke: 2w-12w	Type of vessels: MCA	CCD=3	No follow-up	^{123}I-IMP	SPECT

J.NUUTINEN 2000	n=20, M 7, F 13	70(45-86)	Cross-sectional	Finland, Europe	6	No NHISS admission	After the onset of Stroke: ≤24h	Type of vessels: MCA	CCD=10, CCD=14 (3d)	3d Follow-up	^{99m}Tc-HMPAO+^{99m}Tc-ECD	SPECT
Nobuhiko MIYAZAWA 2001	n=30, M 24, F 6	56.5(36-71)	Cross-sectional	Japan, Asia	8	No NHISS admission	After the onset of Stroke: 41.5±21.3d	Type of vessels: ICA/MCA	CCD=18	No follow-up	^{123}I-IMP	SPECT
Thomas G.Brott 1986	n=30	Unknown	Cross-sectional	USA, North America	5	No NHISS admission	After the onset of Stroke: 72h-40d	Type of vessels: Supratentorial	CCD=15	No follow-up	^{123}I-HIPDM	SPECT
J V Bowler 1992	n=18, M 6, F 12	71.06±11.36	Cross-sectional	UK, Europe	5	No NHISS admission	After the onset of Stroke: 11.9d/ ≤4d	Type of vessels: Lacunar	CCD=6	No follow-up	^{99m}Tc-HMPAO	SPECT
Y Sakashita 1993	n=14, M 8, F 6	76.2±7.3	Cross-sectional	Japan, Asia	6	No NHISS admission	After the onset of Stroke: 5d-22d	Type of vessels: MCA/ACA	CCD=11, ITD=12	No follow-up	^{123}I-IMP	SPECT
Jean-Luc Moretti 1989	n=10, M 6, F 4	65.8±12	Cross-sectional	France, Europe	5	No NHISS admission	After the onset of Stroke: 2d-97d	Type of vessels: MCA	CCD=7	No follow-up	^{99m}Tc-HMPAO+^{123}I-IMP	SPECT
Cheng-Chiang Chang 2017	n=42	Unknown	Cross-sectional	China, Asia	8	No NHISS admission	After the onset of Stroke: 0.5y-1.5y	Type of vessels: Supratentorial	CCD=28	No follow-up	^{99m}Tc-ECD	SPECT
Anna Nocuń 2013	n=57, M 33, F 24	65±19.5	Cross-sectional	Poland, Europe	7	No NHISS admission	After the onset of Stroke: 2m-15m	Type of vessels: Supratentorial	CCD=20	No follow-up	^{99m}Tc-ECD	SPECT
Marc Rousseaux 1999	n=25, M 16, F 9	59.6±12.6	Cross-sectional	France, Europe	8	No NHISS admission	After the onset of Stroke: ≥2w	Type of vessels: Cerebellar	CCD=7	No follow-up	^{99m}Tc-HMPAO+^{133}Xe	SPECT
W.Y.LIN 1996	n=12, M 9, F 3	67.25±6.17	Cross-sectional	China, Asia	6	No NHISS admission	After the onset of Stroke: ≤1d	Type of vessels: MCA	CCD=9	1w/2w/3w follow-up	^{99m}Tc-HMPAO	SPECT
J.Frits de Bruïne 1990	n=26	Unknown	Retrospective cohort	USA, North America	5	No NHISS admission	After the onset of Stroke: ≤24h	Type of vessels: MCA	CCD=18	No follow-up	^{201}TI-DDC	SPECT
M.I.Botez 1991	n=25, M 15, F 10	45.76±14.75	Case-control	Canada, North America	6	No NHISS admission	After the onset of Stroke: ≥1year	Type of vessels: Cerebellar	Cerebello-cerebral Diaschisis=17	No follow-up	^{99m}Tc-HMPAO	SPECT
Ryoo Yamamoto 2015	n=12, M 11, F 1	72.42±8.97	Retrospective cohort	Japan, Asia	6	No NHISS admission	After the onset of Stroke: ≤2w	Type of vessels: Small vessels	CCD=7	No follow-up	^{99m}Tc-ECD	SPECT
Daniela Perani 1988	n=19, M 14, F 5	58.3±11.9	Retrospective cohort	Italy, Europe	7	No NHISS admission	After the onset of Stroke: 2d-24m	Type of vessels: Small vessels	CCD=9	No follow-up	^{123}I-IMP	SPECT

Cong Xia 2021	n=85, M 65, F 20	52.2±11.4	Retrospective cohort	China, Asia	7	NHISS admission: 8(5.5-12)	After the onset of Stroke: 6h-3d,3d-7d,7d-30d	Type of vessels: MCA	ITD+CCD=73	No follow-up		ASL MRI
Shuai Chen 2014	n=46, M 34, F 12	56.89±10.42	Cross-sectional	China, Asia	5	No NHISS admission	After the onset of Stroke: 1w-3w	Type of vessels: Supratentorial	CCD=24	No follow-up		ASL MRI
Koung Mi Kang 2017	n=32, M 21, F 11	62.6±14	Cross-sectional	Korea, Asia	8	NHISS admission: 4.5(2-13)	After the onset of Stroke: ≤8h	Type of vessels: Supratentorial	CCD=24	No follow-up		ASL MRI
Megan K.Strother 2016	n=18, M 7, F 11	47.8±16.1	Cross-sectional	USA, North America	7	No NHISS admission	After the onset of Stroke: ≥2m	Type of vessels: Supratentorial	CCD=9	No follow-up		ASL MRI
Jianhong Ma 2021	n=74, M 47, F 27	62.86±11.79	Cross-sectional	China, Asia	8	No NHISS admission	After the onset of Stroke: 6h-72h	Type of vessels: Supratentorial	CCD=35	No follow-up	Gd-DTPA	DSC-PWI
Alex Förster 2014	n=39, M 20, F 19	72(63-79)	Retrospective cohort	Germany, Europe	6	No NHISS admission	After the onset of Stroke: ≤1d	Type of vessels: Small vessels	CCD=9	No follow-up	Contrast bolus	DSC-PWI
D.D.M.Lin 2009	n=301	Unknown	Retrospective cohort	USA, North America	6	No NHISS admission	After the onset of Stroke: ≤5d	Type of vessels: Supratentorial	CCD=47	No follow-up		DSC-PWI
R Slater 1977	n=15, M 9, F 6	66(47-85)	Retrospective cohort	USA, North America	6	No NHISS admission	After the onset of Stroke: ≤4d	Type of vessels: ICA/MCA/ACA	Transhemispheric Diaschisis=12	No follow-up	^{133}Xe	XeCT
Giorgio Rubin 2000	n=23, M 10, F 13	69±14	Cross-sectional	USA, North America	6	NHISS admission: 14±4	After the onset of Stroke: ≤8h	Type of vessels: MCA	Ipsilateral cerebellar Diaschisis=7	No follow-up	Xe	XeCT
Martina Sebök 2018	n=25, M 18, F 7	57±15	Cross-sectional	Switzerland, Europe	7	NHISS admission: 7(5)	After the onset of Stroke: ≥1week	Type of vessels: ICA/MCA	CCD=10	No follow-up	$^{15}O-H_2O$	PET
Hans L.Lagrèze 1987	n=7, M 7, F 0	64.86±9.93	Cross-sectional	USA, North America	6	No NHISS admission	After the onset of Stroke: 6d-47d	Type of vessels: MCA	Transhemispheric Diaschisis=6	No follow-up	$^{18}FCH_3$	PET
Szilágyi G 2011	n=10, M 7, F 3	59.4±7.2	Cross-sectional	Hungary, Europe	8	No NHISS admission	After the onset of Stroke: 7m-76m	Type of vessels: MCA	CCD=4	No follow-up	^{15}O-butanol/18FDG	PET
Makoto Tanaka 1992	n=12, M 7, F 5	59.5±14.86	Cross-sectional	Japan, Asia	6	No NHISS admission	After the onset of Stroke: ≥1m	Type of vessels: Small vessels	CCD=6	No follow-up	$^{15}O/C^{15}O_2$	PET
J.De Reuck 1997	n=28, M 23, F 5	64(27-79)	Retrospective cohort	Belgium, Europe	6	No NHISS admission	After the onset of Stroke: 4m-24m	Type of vessels: MCA	CCD=8	No follow-up	^{15}O /$C^{15}O_2$	PET
Vince I Madai 2011	n=25, M 13, F 12	58.44±11.81	Retrospective cohort	Germany, Europe	7	NHISS admission	After the onset of Stroke: more	Type of vessels:	CCD=22	No follow-up	^{15}O -H_2O	PET

						n: 9.48±5.97	≤48h, less192h-900h	MCA				
P.PANTANO 1986	n=55, M 29, F 26	61±14	Cross-sectional	Italy, Europe	8	No NHISS admission	After the onset of Stroke: 2d-3y	Type of vessels: ICA	CCD=29	No follow-up	^{15}O /$C^{15}O_2$	PET
Hiroshi Yamauchi 1994	n=11, M 8, F 3	61.8±3	Cross-sectional	Japan, Asia	6	No NHISS admission	After the onset of Stroke: ≥1m	Type of vessels: ICA/MCA	CCD=10	No follow-up	^{15}O /$C^{15}O_2$	PET
S Pappata 1990	n=15	Unknown	Cross-sectional	France, Europe	5	No NHISS admission	After the onset of Stroke: 10d-65d	Type of vessels: Small vessels	Ipsilateral Cortical Diaschisis=3, CCD=5	No follow-up	$^{15}O/C^{15}O_2/^{18}FDG$	PET
Jeffrey A 1989	n=19	61.6±4.1	Case-control	USA, North America	7	No NHISS admission	After the onset of Stroke: 5d-65d	Type of vessels: Unilateral Supratentorial	Transhemispheric Diaschisis=14	No follow-up	$^{18}FCH_3$	PET
Hiroyuki Miura 1994	n=35, M 24, F 11	62.9±7.2	Cross-sectional	Japan, Asia	6	No NHISS admission	After the onset of Stroke: 1d-2336d	Type of vessels: MCA	CCD=31, CCD=23 (5y)	5y Follow-up	^{15}O	PET
Jacques De Reuck 1995	n=28, M 23, F 5	64(27-79)	Case-control	Belgium, Europe	6	No NHISS admission	After the onset of Stroke: 4m-24m	Type of vessels: MCA	CCD+ITD=17	No follow-up	^{15}O /$C^{15}O_2$	PET
Klaus Tatsch 2003	n=11, M 4, F 7	55.8±6.7	Case-control	Germany, Europe	6	No NHISS admission	After the onset of Stroke: Chronic	Type of vessels: Small vessels	CCD=6	No follow-up	^{18}FDG	PET
Carlo Serrati 1994	n=16, M 8, F 8	73.4±7.2	Case-control	France, Europe	7	No NHISS admission	After the onset of Stroke: 5h-30h	Type of vessels: MCA	CCD=9, CCD=4 (follow up)	13d-56d follow-up	^{15}O /$C^{15}O_2$	PET
Martina Sebök 2020	n=24, M 21, F 4	68±10	Prospective cohort	Switzerland, Europe	8	No NHISS admission	After the onset of Stroke: ＞7d	Type of vessels: ICA	CCD=8	No follow-up		fMRI
Lita von Bieberstein 2020	n=25, M 19, F 6	65(33-81)	Retrospective cohort	Switzerland, Europe	6	NHISS admission: 3(5)	After the onset of Stroke: Subacute	Type of vessels: ICA	CCD=11	No follow-up		fMRI
C.H.B.van Niftrik 2021	n=17, M 13, F 4	58.3±12.9	Case-control	Switzerland, Europe	7	No NHISS admission	After the onset of Stroke: ＞3w	Type of vessels: ICA/MCA	ITD=9, CCD=7	No follow-up		fMRI
